# Efficient implementation of the ‘non-biopsy approach’ for the diagnosis of childhood celiac disease in the Netherlands: a national prospective evaluation 2010–2013

**DOI:** 10.1007/s00431-021-04068-1

**Published:** 2021-04-15

**Authors:** Caroline R. Meijer, Joachim J. Schweizer, Anne Peeters, Hein Putter, M. Luisa Mearin

**Affiliations:** 1 Department of Paediatrics, Willem Alexander Children’s Hospital, Leiden University Medical Centre, Albinusdreef 2, 2333 ZA Leiden, The Netherlands; 2 Department of Medical Statistics, Leiden University Medical Centre, Leiden, The Netherlands

**Keywords:** Implementation, Guideline, Childhood celiac disease, Incidence, Clinical presentation

## Abstract

The aim of this study was (1) to prospectively evaluate the nationwide implementation of the ESPGHAN-guidelines for the diagnosis of celiac disease (CD), (2) to investigate the incidence and clinical presentation of diagnosed childhood CD (0–14 years) in the Netherlands, and (3) to compare the findings with national survey data from 1975 to 1990 and 1993 to 2000 using the same approach. From 2010 to 2013, all practicing paediatricians were invited to report new celiac diagnoses to the Dutch Pediatric Surveillance Unit. Data were collected via questionnaires. A total of 1107 children with newly diagnosed CD were reported (mean age, 5.8 years; range, 10 months–14.9 years; 60.5% female). After the introduction of the non-biopsy approach in 2012, 75% of the diagnoses were made according to the guideline with a significant decrease of 46.3% in biopsies. The use of EMA and HLA-typing significantly increased with 25.8% and 62.1%, respectively. The overall incidence rate of childhood CD was 8.8-fold higher than in 1975–1990 and 2.0-fold higher than in 1993–2000. During the study period, the prevalence of diagnosed CD was 0.14%, far below 0.7% of CD identified via screening in the general Dutch paediatric population. Clinical presentation has shifted towards less severe and extra-intestinal symptoms.

*Conclusion*: ESPGHAN guidelines for CD diagnosis in children were effectively and rapidly implemented in the Netherlands. Incidence of diagnosed CD among children is still significantly rising with a continuous changing clinical presentation. Despite the increasing incidence of diagnoses, significant underdiagnosis still remains.

**Table:** 

**What is Known:** *• Since 2000 the incidence of diagnosed childhood CD in the Netherlands has shown a steady rise.* *• The rise in incidence has been accompanied by a changing clinical presentation at diagnosis.*	
**What is New:** *• The ESPGHAN guidelines 2012 for CD diagnosis were effectively and rapidly implemented in the Netherlands.* *• The incidence of diagnosed childhood CD in the Netherlands has continued to rise significantly during the reported period.*

**Supplementary Information:**

The online version contains supplementary material available at 10.1007/s00431-021-04068-1.

## Introduction

Celiac disease (CD) is an immune-mediated systemic disorder elicited by gluten and related prolamins in genetically susceptible individuals and characterised by the presence of a variable combination of gluten-dependent clinical manifestations, CD-specific antibodies, HLA-DQ2 or HLA-DQ8 haplotypes, and enteropathy [1]. Up until a few decades ago, CD was considered an uncommon disease that mainly affected children and limited to Western Europe. However, the current prevalence of CD in the general population is estimated to be approximately 1% in different parts of the world [2, 3].

In the Netherlands, two national surveys on CD diagnosed in childhood performed by our group between 1975 and 1990 (retrospective) and 1993 and 2000 (prospective) showed that the incidence of diagnoses increased significantly from 0.18/1000 to 0.81/1000 live births, respectively [4, 5]. However, as also reported in other countries [6, 7], this increase in the incidence of diagnoses did not correspond nearly as much with the prevalence of CD detected by screening in the overall paediatric population [8, 9], indicating that CD was heavily underdiagnosed in the Netherlands. Our previous Dutch surveys showed that the clinical presentation in children had also shifted towards more subtle symptoms [4, 5]. The results of our prospective study from 1993 to 2000 were based on data from the Dutch Pediatric Surveillance Unit (DPSU) comprising all Dutch paediatric practices, with a mean response rate of 90% (2010). The CD diagnoses were cross-checked by reviewing the National Database of Pathology (in Dutch: Pathologisch Landelijk Geautomatiseerd Archief–PALGA), to identify all biopsy-proven CD cases according to the European Society for Pediatric Gastroenterology, Hepatology and Nutrition (ESPGHAN) 1990 diagnostic criteria [10]. In 2012, ESPGHAN published new diagnostic guidelines with the so-called non-biopsy diagnostic approach for symptomatic children suspected for CD [1]. Nevertheless, novel diagnostic guidelines are not always effectively implemented in daily practice [11].

The aims of the present study are to (i) prospectively evaluate the nationwide implementation of the ESPGHAN guidelines 2012 for CD diagnosis in the Netherlands and (ii) investigate the incidence and clinical presentation of diagnosed childhood CD from 2010 to 2013 in the Netherlands in comparison to previous national surveys.

## Methods

A four-year prospective observational cohort study, including all children aged 0 -14 years and diagnosed with CD throughout the Netherlands between January 1^st^, 2010 and December 31^st^, 2013 as reported to the DPSU. The purpose of the DPSU of the Dutch Society of Pediatrics (DSP) is to gain insight on the prevalence of diseases in youths (0–18 years) on a population level and to promote scientific research addressing the background, nature, prognosis, treatment, and prevention of these diseases [11]. All Dutch paediatricians were asked by paper (until 2010) or through an Internet-based system to report new cases of selected conditions, for our study CD, on a monthly basis, followed immediately or later by completing a questionnaire. This questionnaire, which was filled in by the paediatrician, collected patient information such as gender, age, parents’ country of origin, symptoms at presentation, anthropometrics (height and weight), associated diseases, family history, and (results of) diagnostic tests. Personal data were limited to initials and birth dates to guard patient confidentiality. The completed questionnaires were subsequently sent to the investigators of the Leiden University Medical Centre (LUMC) where the data were stored and analysed. In December 2013, registration was unintentionally closed due to relocation of the DPSU to another organisation. Up until 2012, data from the DPSU were cross-checked using information provided by PALGA, the database that anonymously registers all pathological specimens collected in the Netherlands (including sex, age, date of biopsy). The primary outcome comprised the diagnostic work-up before and after the introduction of the non-biopsy diagnostic approach in 2012. The secondary outcome was the clinical presentation compared to that from previous surveys and the incidence of diagnosed CD in the Netherlands from 2010 to 2013 in children aged 0–14 years as the numerator and the number of live-births in these years as the denominator, expressed as a rate per 1000 live births. The age of the included children (0–14 years) and the metrics were chosen with the purpose to be able to compare the results to those reported in our previous surveys.

Patient information was completely anonymised and guaranteed throughout the study.

The ethical aspects have been approved by the DPSU of the DSP in accordance with the applicable rules on privacy. According to Dutch Law for the use of completely anonymous data, informed consent is not needed.

### Statistical analysis

All categorical data are described as frequencies. Percentages are based on the total number of included patients.

For the incidence rate, we used the data from all the reported children, and for the analysis of the clinical picture and the diagnostic work-up, we used the data from the children with completed questionnaires. Demographic and epidemiological data regarding the general population were provided by the Dutch Central Bureau of Statistics (CBS, The Hague, the Netherlands) [12]. The emigration and immigration rates per 1000 inhabitants in the Dutch population remained stable during the study period (2010 and 2013: 1.2 and 1.1) [13].

The diagnostic approach, incidence rates, and clinical presentation of CD in 2010–2013 were compared to the data from 1975 to 1990 and 1993 to 2000 using the Chi-square test and Chi-square test for trend. A *p*-value of 0.05 was considered statistically significant. Statistical analyses were performed using SPSS 23.0.

## Results

From January 1, 2010, to December 31, 2013, 1325 children with CD were reported to the DPSU, 218 of which were excluded (78 older than 15 years at diagnosis, 123 double reported, 11 withdrawn by paediatrician, 6 diagnosed outside the study period). Of the 1107 included patients (mean age, 5.8 years; range, 10 months–14.9 years; 60.5% female), 209 were only reported as new CD diagnosis, and from the additional, 898 completed questionnaires were returned. The mean survey response rate of Dutch paediatricians to the monthly CD request was 81.1%, of which 87.1%, 84.7%, 77.4%, and 74.1% pertained to the years 2010, 2011, 2012, and 2013, respectively.

### Diagnostic approach

The diagnostic approach is summarised in Table 1. Utilisation of the anti-gliadin antibodies (AGA) and endomysium antibodies (EMA) tests decreased significantly over the period 1993–2000 and 2010–2013 from 90 (*n*=915) to 9.4% (*n*=84) (*p*<0.001) and from 78.0 (*n*=793) to 60.5% (*n*=543) (*p*<0.001), respectively. In contrast, the use of the EMA test increased from 48.7 (*n*=237) in 2010–2011 to 74.5% (*n*=306) in 2012–2013 (*p*<0.001). This was also the case for HLA typing which increased significantly from 23.8 (*n*=116) in 2010–2011 to 85.9% (*n*=353) in 2012–2013 (*p*<0.001). Anti-tissue transglutaminase antibody (tTG) levels were determined in the majority of children (96.8%, *n*=869) diagnosed in 2010–2013. Moreover, in this last period, 66.9% (*n*=601) children underwent diagnostic small bowel biopsies which showed a significant decrease from 88.1 (*n*=429) to 41.8% (*n*=172) (*p*<0.001) after the publication of the non-biopsy ESPGHAN guideline in 2012 [1] (Table 1).

**Table 1:** Changing diagnostic work-up for celiac disease in children in the Netherlands

Diagnostic tests No. (%)	National surveys			2010–2013			
	1975–1990 Retrospective	1993–2000 Prospective	2010–2013 Prospective	2010–2011		2012***–2013	
	*n*=223	*n*=1017	*n* = 898	*n* = 487		*n* = 411	
				Sympt. *n*=454	Asympt. *n*=33	Sympt. *n*=384	Asympt. *n*=27
IgA AGA	131 (59)	915 (90)	84 (9.4)*	45 (9.9)	0	39 (10.2) ^n.s.^	0 ^n.s.^
				45 (9.2)		39 (9.5) ^n.s.^	
IgA tTG	N.A.**	N.A.**	869 (96.8)	440 (96.9)	33 (100)	370 (96.4) ^n.s.^	26 (96.3) ^n.s.^
				473 (97.1)		396 (96.4) ^n.s.^	
IgA EMA	Unknown	793 (78)	543 (60.5)*	223 (49.1)	14 (42.4)	288 (75.0)^	18 (66.7)^
				237 (48.7)		306 (74.5)*	
HLA typing	Unknown	Unknown	469 (52.2)	107 (23.6)	9 (27.3)	329 (85.7)^	24 (88.9)^
				116 (23.8)		353 (85.9)^	
Biopsies	223 (100)	1017 (100)	601 (66.9)*	399 (87.9)	30 (90.1)	150 (39.1)^	22 (81.5)^n.s.^
				429 (88.1)		172 (41.8)^	

On the left side, the data from the three national surveys are presented (1975–2013), and on the right side, the data before and after the introduction of the non-biopsy approach

*p<0.01; *NA* not available at the time

**Widespread introduction throughout the Netherlands in 1999

***Publication of ESPGHAN Guideline. Comparison of data between 2010 and 2011 and 2012 and 2013: n.s., not significantly different or ^ significantly different

In total, 411 children were newly diagnosed with CD in 2012–2013. From them 93.4% (*n*=384) was symptomatic, and 6.6% (*n*=27) was asymptomatic. Two hundred thirty-four of the symptomatic children had tTG levels ≥10x upper limit of normal (ULN) and were eligible for the non-biopsy approach; more than 75% (58/234) of the children were correctly diagnosed according to the guideline. Of all symptomatic children, 77.3% (297/384) were correctly diagnosed as well as 81.5% (22/27) of the asymptomatic children. So, a total of 77.6% of the children (319/411) were correctly diagnosed according to the new ESPGHAN algorithms. Reasons for incorrect application of the ESPGHAN guidelines of 2012 regarding the symptomatic algorithm (in which data were missing for 2 children) were presence of Marsh classification score of 0–1 (*n* = 11; 12.6%) and include missing EMA, HLA-typing, and tTG-tests in 46 (52.9%), 21 (24.1%), and 7 (8.0%) children, respectively. In 5 children, the reasons for incorrect application of the asymptomatic algorithm (in which data were missing for 2 children) were refusal to undergo diagnostic biopsies (*n* = 3; 60.0%).

### Frequency rates

**Fig. 1:**
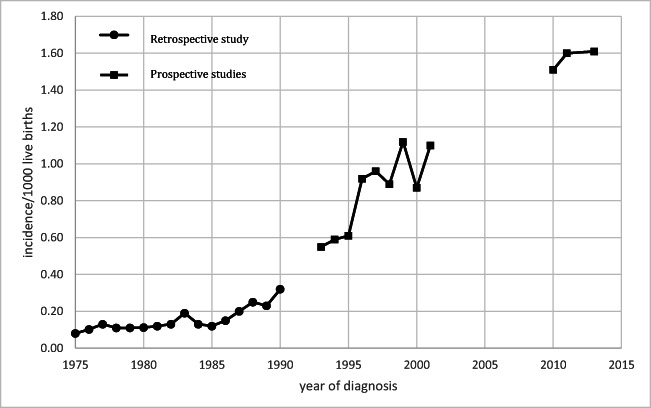
Incidence of diagnosed childhood celiac disease in three national studies in the Netherlands (*n*=223 in 1975–1990; *n*=1017 in 1993–2000, and *n*=1107 in 2010–2013)

Figure 1 details the significantly higher crude incidence rate of diagnosed CD in 2010–2013 (1.59/1000 live births) as compared to the previous studies from 1975 to 1990 and 1993 to 2000, which report incidences of 0.18 and 0.81 per 1000 live births, respectively [4, 5] (*p*<0.001). The reported crude incidence rate of diagnosed CD in the present study was 1.51 per 1000 live births in 2010, 1.60 in 2011, 1.86 in 2012, and 1.35 in 2013 (Fig. 1). The prevalence of diagnosed CD in 2010–2013 was 0.14%, which is significantly lower than the 0.5% detected by screening of the general Dutch paediatric population of 2–4-year-olds reported in 1999 and 0.7% of 6-year-olds reported in 2015 (*p*<0.001) [8, 9].

### Clinical presentation

Characteristics of the reported CD patients are presented in Supplementary Table 1. Parents of 92.2% of children (*n*=828) reported one or more CD-related symptoms at the time of diagnosis, with abdominal pain, wasting (defined as weight <p10) and stunting (height for age < p10) being the most frequently reported symptoms at 49.6% (*n*=445), 33.9% (*n*=304), and 32.0% (*n*=287), respectively (Supplementary Table 1). Only 36 patients (4.0%) presented with the classical triad, i.e. chronic diarrhoea, abdominal distension, and failure to thrive. At least 1 gastrointestinal symptom was reported in 669 (74.5%) patients, while 149 (16.6%) exclusively experienced extra-intestinal symptoms.

Table 2 shows the continuous and significantly changing clinical presentation of diagnosed CD in comparison to the presentation reported in 1975–1990 and in 1993–2000. Although there is a significant decrease in chronic diarrhoea and abdominal distension as presenting symptoms, significantly more children presented with abdominal pain, lassitude, and anorexia. Thirty percent of the children were ≤ 2 years of age, which was significantly younger than reported by the previous surveys. In total, 13.8% of the children had a first-degree relative (FDR) with CD, while only 7.0% of them were referred to the paediatrician for screening based on a positive family history for CD.

**Table 2:** Changing diagnostic work-up for celiac disease in children in the Netherlands

Characteristics	1975–1990 % (n=223)	1993–2000 % (n=1017)	2010–2013 % (n=898)	P value
Chronic diarrhoea	72	41	25	<0.01
Abdominal distension	76	48	28	<0.01
Growth failure in height and weight	63	24	19	<0.01
Weight for height < P10	22	49	34	<0.01
Height for age < P10	42	34	32	<0.01*
Abdominal pain	7	16	50	<0.01
Lassitude	Not known	12	24	<0.01
Anorexia	0	5	24	<0.01
Age ≤ 2 yr.	60	47	30†	<0.01
Median age (yr.)	1.5	2.1	5.8†	<0.01

* Comparison of data only significantly different between 1975–1990 and 2010–2013

† Age of all 1107 reported CD children

## Discussion

In 2012, ESPGHAN published new guidelines for the diagnosis of CD in children and adolescents, including the novel so-called non-biopsy approach for selected cases [1]. Our national prospective data show that in 2012–2013, childhood CD was diagnosed in the Netherlands according to the new guidelines in more than 75% of the cases, with 75.2% correct application of the ‘non-biopsy’ approach, indicating a quick and efficient implementation of the new guidelines. Such successful implementation does not always follow the publication of novel guidelines [11]. For example, after the publication of the guideline for the diagnosis and management of gastroesophageal reflux in children, only 1.8% of the general paediatricians showed complete adherence to it [11], a frequency that increased to 46.1% after specific training [14].

The effective implementation in the Netherlands has possibly been facilitated first because they were actively overtaken by the DSP immediately following their publication and second because of the extended use of the highly sensitive tTG-test which is imperative in the 2012 ESPGHAN diagnostic guidelines [1, 15] (Table 1). The variable use of the EMA-test, both in the Netherlands and in other countries [16], is explained by the introduction of the more simple and economical tTG-test in the 1990s, followed by an increase in its use after the publication of the ESPGHAN guidelines of 2012 in which its determination was established for the initial diagnostic work-up and for the confirmation of CD diagnoses under the non-biopsy approach [1, 17, 18]. The use of the EMA-test as a confirmatory diagnostic test has been reinforced by the updated ESPGHAN guidelines of 2020, so an increase in its implementation may be expected in the future, particularly in children diagnosed without biopsies [19]. The significant reduction (46.3%) of diagnostic biopsies in our country, which is in accordance with findings from other studies [16, 20], indicates that the implementation of the non-biopsy strategy has taken place quickly and efficiently. However, the guidelines for non-biopsy diagnosis in children have not yet been adopted in all countries, despite its numerous advantages such as the reduction in medical costs and avoidance of general anaesthesia or deep sedation [21, 22].

With the conditional recommendation of the non-biopsy approach in the ESPGHAN guidelines of 2020 for asymptomatic children, a further decline in small bowel biopsies is to be expected.

Our data show a continuous and significant 8.8-fold increase in the incidence of CD diagnosed in childhood in the Netherlands from 1975 to 2013, with a 2.0-fold increase from 1993 to 2000 to 2010 to 2013. This is in accordance with the 2.4-fold increase found in the retrospective nationwide survey on newly diagnosed CD both in children and adults from 1995 to 2010 [23]. Our results also agree with the findings from recent European and Canadian studies conducted in paediatric populations which likewise show an increasing trend over time in the frequency of clinically diagnosed childhood CD [24–26]. The rising incidence in the number of diagnoses is likely caused by a combination of several factors, namely, the growing awareness of CD among healthcare professionals, increased screening of high-risk groups, and the availability of reliable CD antibody tests [1], but also a true rise in the incidence of CD [27]. In this respect, an increasing prevalence of CD has been shown in screening studies among school-aged children with a 1.4-fold increase over a period of 15 years in the Netherlands and over 1.8 times in 25 years in Italy [8, 9, 28]. This is in line with the 5-fold increase in prevalence of CD autoimmunity over a period of 50 years found in the USA, a finding based on the analysis of stored sera from community subjects compared with sera collected at an earlier date [29]. In contrast, no increase over time in the prevalence of CD has been reported in adult blood donors in Israel [30].

Strengths of our study include the reporting of national data which forms a seamless representation for the whole country of the Netherlands, as well as utilisation of the same methods as in the survey performed in 1993–2000, improving the reliability of the result comparisons. Nevertheless, a possible limitation of our study is the decreased response rate to the DPSU monitoring system (from 87.1 in 2010 to 74.1% in 2013 versus 90% in 1993–2000) [5]. This decrease is possibly due to the overall increasing administrative burden, complexity of care, and reduced time for reporting among Dutch paediatricians as well as the relocation of the DPSU to another organisation at the end of 2013 [31]. The relatively low response rate of 2013, which does not represent a true decline in the incidence of CD diagnoses, is the most plausible cause of the abrupt decrease in the incidence of CD diagnoses reported to the DPSU in this year when compared to previous years, even after correcting for the preliminary closing of the reporting system.

Our findings of a continuously changing clinical presentation and significant increase in the median age at diagnosis are in agreement with those reported by other countries [16–19, 32–38]. The actual clinical presentation of CD diagnosed in childhood in the Netherlands is formed by a variable combination of abdominal pain and poor growth (in weight or in height). The classical triad of diarrhoea, abdominal distension, and failure to thrive is rare although each of these symptoms is present in many CD patients (Supp Table 1) [39, 40]. Failure to thrive (defined as height for age <p10 and weight for height <p10) occurs significantly less frequent than before (44%), even though the absolute frequency has remained fairly stable over the time (*n*=140 in 1975–1990, *n*=244 in 1993–2000, and *n*=166 in the present study). Interestingly, 70% of the diagnosed children in 2010–2013 had at least one non-gastrointestinal symptom, with lassitude and anorexia also increasing significantly (Table 1) [4, 5]. The shift in CD presenting symptoms towards a milder form of disease may also potentially be the reason for an upward shift of age at diagnosis [39, 40].

In conclusion, the ESPGHAN guidelines 2012 for the diagnosis of CD in children were effectively and quickly implemented in the Netherlands. During the 2 years after their publication, the guidelines were applied in more than 75% of the cases, particularly in older children. The clinical presentation of childhood CD in the Netherlands is characterised by a continuous change with a shift towards less severe and non-gastrointestinal symptoms. The incidence of diagnosed CD in childhood from 2010 to 2013 in the Netherlands has increased significantly by 8.8-fold from 1975 to 1990 and 2.0-fold from 1993 to 2000. Despite the rising incidence in the number of diagnoses, the prevalence of diagnosed CD is significantly lower that the prevalence of disease identified by screening, signifying that childhood CD is still significantly underdiagnosed in the Netherlands.

## Supplementary information

ESM 1 (DOCX 21 kb)

## Data Availability

Available.

## Abbreviations

AGA: Anti-gliadin antibodies
CBS: Central Bureau of Statistics
CD: Celiac disease
DPSU: Dutch Pediatric Surveillance Unit. In Dutch: Nederlands Signalerings Centrum Kindergeneeskunde, NSCK
DSP: Dutch Society of Paediatrics
EMA: Endomysium antibodies
ESPGHAN: European Society for Pediatric Gastroenterology Hepatology and Nutrition
FDR: First-degree relative
HLA: Human leukocyte antigen
LUMC: Leiden University Medical Centre
PALGA: Pathologisch Landelijk Geautomatiseerd Archief–National Database of Pathology
tTG: Anti-tissue transglutaminase antibodies
ULN: Upper limit of normal
