# Supplementary material for: Efficient implementation of the ‘non-biopsy approach’ for the diagnosis of childhood celiac disease in the Netherlands: a national prospective evaluation 2010–2013
Source: Eur J Pediatr. 2021 Apr 15;180(8):2485–92. doi: 10.1007/s00431-021-04068-1 (PMC8285331; doi:10.1007/s00431-021-04068-1)
Supplement: Supplementary file 1 — (DOCX 21 kb) [file 431_2021_4068_MOESM1_ESM.docx]

**Supplementary table 1. Characteristics of 898 children with celiac disease**

**diagnosed in 2010-2013 as reported to the Dutch Paediatric Surveillance Unit**

|  | **2010**  **n = 243** | **2011**  **n = 244** | **2012**  **n = 254** | **2013**  **n = 157** | **Total**  **n = 898** |
| --- | --- | --- | --- | --- | --- |
| **Median age at diagnosis of CD, in years**  **Age ≤ 2, in %** | 5.7  29.7 | 6.0  28.5 | 5.8  32.0 | 6.0  29.2 | **5.8** |
| **Female, No. (%)**  **Unknown** | 166 (68.3)  6 (2.5) | 159 (65.2)  12 (4.9) | 139 (54.7)  22 (8.7) | 79 (50.3)  21 (13.4) | **543 (60.5)**  **61 (6.8)** |
| **Reason for referral, No. (%)**  Suspected CD  Positive family history  Associated disease  Suspected CD + positive family history  Suspected CD + associated disease  Unknown | 188 (77.4)  18 (7.4)  20 (8.2)  2 (0.8)  2 (0.8)  13 (5.3) | 201 (82.4)  17 (7.0)  22 (9.0)  0 (0)  0 (0)  4 (1.6) | 209 (82.3)  17 (6.7)  22 (8.7)  0 (0)  0 (0)  6 (2.4) | 121 (77.1)  11 (7.0)  19 (12.1)  2 (1.9)  0 (0)  3 (1.9) | **719 (80.1)**  **63 (7.0)**  **83 (9.2)**  **5 (0.6)**  **2 (0.2)**  **26 (2.9)** |
| **Symptoms, No. (%)**  No symptoms  Anorexia  Recurrent oral ulcers  Nausea  Vomiting  Abdominal pain  Abdominal distension  Constipation  Acute diarrhoea (<15 days)  Chronic diarrhoea (>4 weeks)  Pallor  Lassitude  Irritability  Delayed puberty  Joint disorders  Failure to thrive  Wasting (Weight for height < P10)  Stunting (Height for age < P10)  Non-gastrointestinal symp  Unknown | 20 (8.2)  59 (24.3)  5 (2.1)  8 (3.3)  25 (10.3)  104 (42.8)  69 (28.4)  45 (18.5)  6 (2.5)  61 (25.1)  28 (11.5)  55 (22.6)  37 (15.2)  2 (0.8)  3 (1.2)  41 (16.9)  79 (32.5)  74 (30.5)  191 (68.5)  13 (5.3) | 23 (9.4)  53 (21.7)  4 (1.6)  10 (4.1)  32 (13.1)  113 (46.3)  69 (28.3)  46 (18.9)  7 (2.9)  55 (22.5)  33 (13.5)  67 (27.5)  41 (16.8)  3 (1.2)  1 (0.4)  47 (19.3)  79 (32.4)  88 (36.1)  186 (64.6)  2 (0.8) | 17 (6.7)  64 (25.2)  1 (0.4)  18 (7.1)  28 (11.0)  137 (53.9)  72 (28.3)  56 (22.0)  7 (2.8)  68 (26.8)  26 (10.2)  65 (25.6)  54 (21.3)  0 (0)  1 (0.4)  50 (19.7)  97 (38.2)  73 (28.7)  238 (72.6)  4 (1.6) | 10 (6.4)  39 (24.8)  1 (0.6)  14 (8.9)  17 (10.8)  91 (58.0)  39 (24.8)  30 (19.1)  8 (5.1)  36 (22.9)  8 (5.1)  28 (17.8)  26 (16.6)  0 (0)  1 (0.6)  28 (17.8)  49 (31.2)  52 (33.1)  161 (75.9)  1 (0.6) | **70 (7.8)**  **215 (23.9)**  **11 (1.2)**  **50 (5.6)**  **102 (11.4)**  **445 (49.6)**  **249 (27.7)**  **177 (19.7)**  **28 (3.1)**  **220 (24.5)**  **95 (10.6)**  **215 (23.9)**  **158 (17.6)**  **5 (0.6)**  **5 (0.6)**  **166 (18.5)**  **304 (33.9)**  **287 (32.0)**  **776 (70.1)***  **18 (2.0)** |
| **Associated disease, No. (%)**  Type 1 Diabetes  Down Syndrome  Turner Syndrome  Selective IgA Deficiency (0.05 g/l)  Other#  Unknown | 28 (11.5)  20 (8.2)  3 (1.2)  1 (0.4)  3 (1.2)  1(0.4)  12 (4.9) | 29 (11.9)  9 (3.7)  15 (6.1)  -  3 (1.2)  2 (0.8)  6 (2.5) | 31 (12.2)  16 (6.3)  12 (4.7)  -  2 (0.8)  1 (0.4)  10 (3.9) | 19 (12.1)  12 (7.6)  5 (3.2)  -  1 (0.6)  1 (0.6)  3 (1.9) | **107 (11.9)**  **57 (6.3)**  **35 (3.9)**  **1 (0.1)**  **9 (1.0)**  **5 (0.6)**  **31 (3.5)** |
| **Relative with CD, No (%)** | 37(15.2) | 31(12.7) | 34 (13.4) | 22(14.0) | **124 (13.8)** |

*149 children had exclusively non-gastrointestinal symptoms. #Rheumatoid Arthritis and Autoimmune Thyroiditis
